# Individually designed fully covered self-expandable metal stents for pediatric refractory benign esophageal strictures

**DOI:** 10.1038/s41598-021-01921-z

**Published:** 2021-11-19

**Authors:** Xing Wang, Haifeng Liu, Zhihong Hu, Rufang Zhang, Zhujun Gu, Kai Lin, Yuling Feng

**Affiliations:** 1 Department of Digestive Endoscopy Center, Shanghai Children’s Hospital, Shanghai Jiao Tong University, No. 355 Luding Road, Shanghai, 200062 China; 2 Department of Cardiothoracic Surgery, Shanghai Children’s Hospital, Shanghai Jiao Tong University, Shanghai, China

**Keywords:** Gastrointestinal diseases, Diseases

## Abstract

To share our institutional experience of placing individually designed fully covered self-expandable metal stents (FCSEMSs) for the treatment of refractory benign esophageal strictures (RBESs) in pediatric patients. A 10-year retrospective study between May 2009 and July 2020 that includes 14 children with RBESs who were treated with individually designed FCSEMSs. Patients were followed-up regularly after stent placement to observe the improvement of vomiting and dysphagia, changes in stenosis diameter and complications. A total of 20 stents were successfully placed in 14 patients. During a follow-up period ranging from 5 to 83 months, except for one 4-year-old child who could not endure chest pain, the remaining 13 patients all benefited from stenting. Their Ogilvie & Atkinson scores improved from grade III–IV to grade 0-I, and the diameters of the stenosis’ were enlarged from 2–5 mm to 9–14 mm. Two patients developed restenosis and granulation tissue hyperplasia was found in 2 patients and stent migration and malapposition in 2 patients with esophageal perforations that required further endoscopic intervention. The use of FCSEMS for RBES is safe and effective in selected pediatric patients. Rationally designed stents and timely management of postoperative complications are critical to ensure the success of this new method.

## Introduction

Refractory benign esophageal strictures (RBESs) refers to benign esophageal stenosis when persistent dysphagia cannot be relieved after repeated surgical interventions^1^. The main causes of RBESs in children include chemical corrosive esophageal injury, achalasia of the cardia, anastomotic stricture after esophagectomy, as well as tracheoesophageal fistula^2^. Traditional treatment methods used to be either esophagoplasty or Savary bougie dilatation^3,4^. However, due to the operational complexity and complications of perforation, respectively both interventions are now only used as the last resort for managing RBESs^5^. Therefore, a more minimally invasive operation like endoscopic balloon dilatation (EBD) has been popularized in dealing with these pediatric disease entities, with evidence that over 80–90% of children with esophageal stenosis achieved clinical remission through this method^6,7^.

Nevertheless, some children with dysphagia showed no significant improvement after repeated balloon dilatation, and recurrent stricture occurred in a very short period of time. For these children, it is very important to find a more safe and effective technique. In 1983, Frimberger^8^ first reported the success of metal stents for treating esophageal stenosis, which provided a new approach for the treatment of esophageal stenosis. With the improvement and refinement of stent technology, more and more stenting procedures have been performed on various esophageal pathologies including RBESs in clinical practice. Nitinol fully covered self-expandable metal stent (FCSEMS) is a type of esophageal stent widely used in recent years for endoscopic therapy of esophageal stenosis in adults^9^. The stent is woven into a network of 0.15 mm nitinol wire, which possesses elasticity, shape memory functions and good biocompatibility^10^. Mechanically, the restoring and elastic force produced by the stent at body temperature produces a long-lasting effect on stenosis of the esophagus, which can expand and keep the esophagus unobstructed. Since this expansion process is gradual, the patient can adapt to it, and the tear of the esophageal mucosa occurs in a deliberate fashion, which effectively reduces the risk of bleeding and perforation. In view of the superiorities of this type of stent, it is anticipated that children with RBESs will be effectively treated by its placement.

As a special treatment group, children are not completely treated as are adults due to their developmental characteristics. As far as we are aware at present, there are neither clear guidelines for the treatment of RBES with an esophageal stent nor specific esophageal stents designed for children^11^. The number of cases of children with RBES treated with stents is relatively small^12^, so this retrospective study was designed to analyze the clinical data of pediatric patients with RBESs who were treated with FCSEMS in our hospital from May 2009 to July 2020, with the aim of exploring the clinical feasibility of FCSEMS for treating pediatric RBES.

## Methods

### Patients

This retrospective study enrolled pediatric patients with the following inclusion criteria: (1) Benign esophageal stricture diagnosed on clinical presentation using upper gastrointestinal tract radiography, computerized tomography (CT) or gastroscopy; (2) Dysphagia symptoms did not improve significantly or the stenosis recurred within a short period after ≥ 3 sessions of balloon dilatation; (3) The severity of dysphagia was graded to be III to IV by following Ogilvie & Atkinson scores^13,14^; (4) Patients were < 18 years old. The exclusion criteria were: (1) Patients with severe cardiopulmonary disease or coagulation dysfunction, who could not tolerate surgical intervention; (2) The medical records of patients did not provide complete case data.

Based on the inclusion and exclusive criteria, 14 consecutive patients were enrolled in the study from May 2009 to July 2020. This retrospective study was approved by the ethics committee of Shanghai Children's Hospital followed the tenets of the Declaration of Helsinki. Written informed consent of the patient was obtained.

### Preoperative preparation

Preoperative upper gastrointestinal tract radiography or CT examinations were performed to assess the location, length and diameter of esophageal stenosis. FCSEMSs were customized according to the specific conditions of the stenotic segment. The patients had fasted for more than 12 h before their operations.

### Stent customization

**Figure 1:**
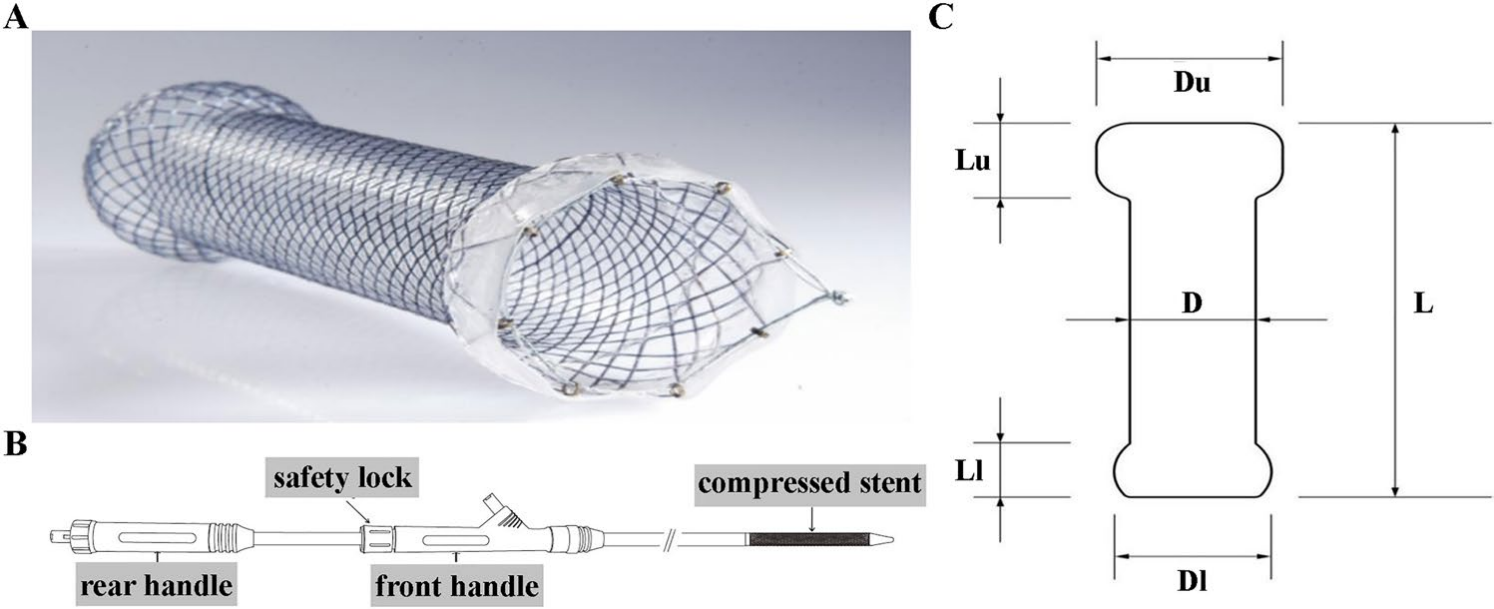
The nitinol fully covered self-expandable metal stents (FCSEMS), the stent delivery device and design, and characterization of this type of stent: Du and Lu are the diameter and length of the upper bell mouth, respectively. Dl and Ll are the diameter and length of the lower bell mouth, respectively.

According to the length of the narrow segment, the length of the stent was further tailored to be 1.5 cm and 1 cm greater than the upper and lower edges of the narrowing, respectively. Next, the diameter of the stent was determined by the size of the inner diameter of the stenosis to avoid excessive compression of the stent to the wall of the feeding tube (Fig. 1). The experience of our practice suggested that the inner diameter of the different narrow segments should be fitted by with commercially available stents with diameters of 12 mm, 14 mm and 16 mm. Simply put, stents with an inner diameter of 12–14 mm were selected for children < 5 years old, while 16 mm stents were more suitable for children > 6 years old or children with an esophageal perforation.

Furthermore, the upper and lower ends of the stents were designed with a bell mouth shape, which was about 6 ± 0.25 mm larger than the inner diameter of the stent, so as to closely fit the upper and lower esophageal wall of the narrow segment. A silicone membrane skirt was used to increase friction to minimize the occurrence of displacement (Fig. 1). However, for the stenosis of the middle and lower segment, especially the stenosis near the second esophageal stenosis, the diameter of the bell mouth on the stent should not be too large because of the thin wall of the esophageal tube in children and its complex anatomical position. Chest CT scan were carried out before the placement operation to determine the position of the esophagus, trachea and aorta, and then a stent with a smaller upper bell mouth was positioned to avoid compression of the trachea and aortic arch, thereby reducing unforeseen cardiovascular risks.

### Stenting procedure

**Figure 2:**
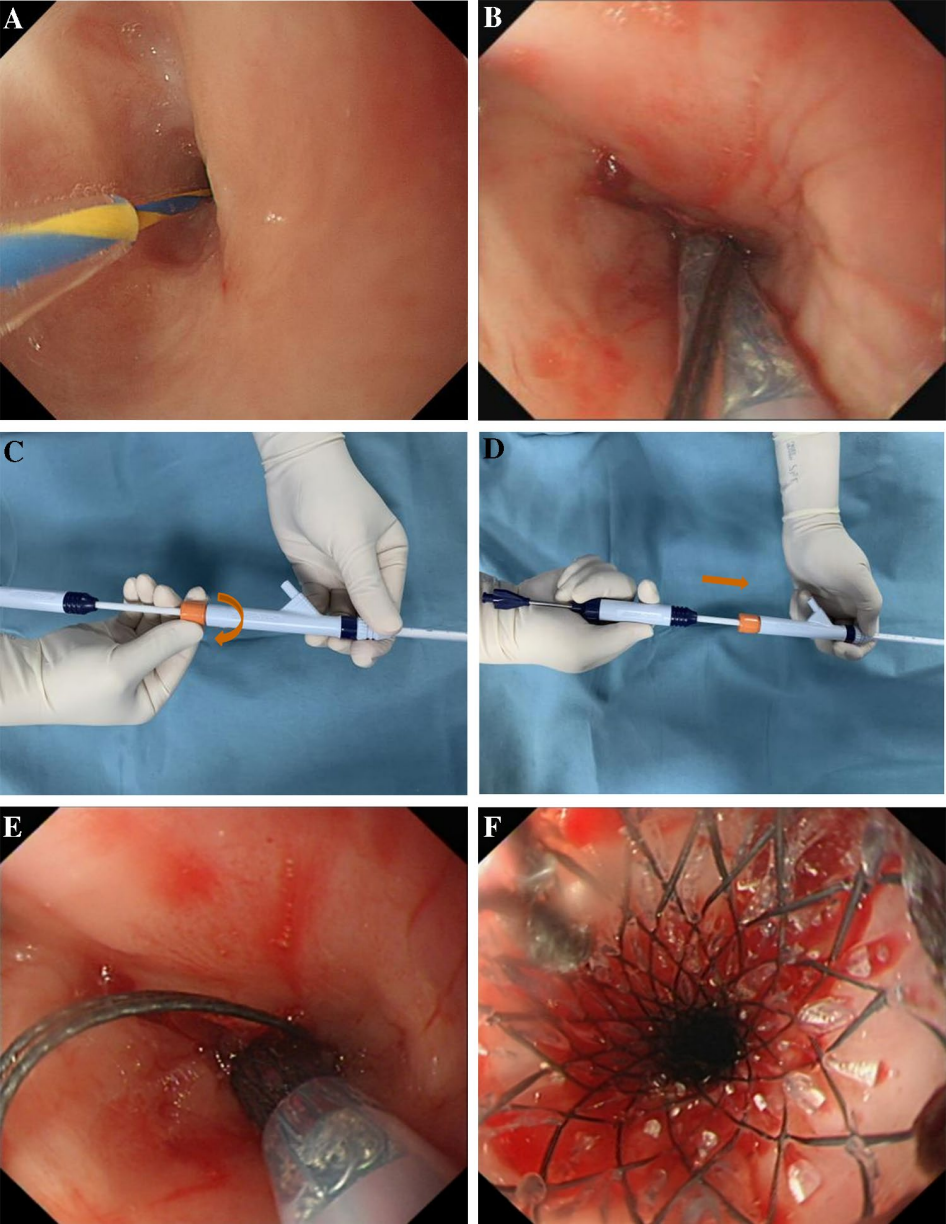
Illustration of fully covered self-expandable metal stent replacement. (**A**) Endoscopic view of the preoperative stricture with the zebra super-hard guide wire inserted into the stomach; (**B**) Under the guidance of the guidewire, FCSEMS was delivered to the stricture site through the stent delivery device; (**C**,**D**) Opening the safety lock and pushing the rear handle forward slowly to release the stent; (**E**,**F**) The stent was released and the lumen enlarged after FCSEMS placement.

Tracheal intubation was performed under general anesthesia, then an electrocardiograph with blood oxygen monitoring was connected to the patients, and the tooth pad was placed afterwards. Under the guidance of an electronic gastroscope (CV260/290, Olympus), a zebra super-hard guidewire (AMH-GW-S3545, Anrei Medical (Hangzhou) Co., Ltd) was inserted into the stomach, the position of the guidewire was retained and then the gastroscope withdrawn. Under the guidance of the guidewire, the stent delivery device (MTN-SR-6/650, Micro-Tech (Nanjing) Co., Ltd) was slowly delivered to the FCSEMS (MTN-SE-S-14/60-A-6/650, Micro-Tech (Nanjing) Co., Ltd) (Fig. 1) of the esophageal stricture. The proximal end of the stent was 10–20 mm higher than the predetermined release position under endoscopy surveillance (Fig. 2). After confirming the position, the safety lock of the device was opened, then the rear handle was fixed with one hand, and the front handle was held and pulled back slowly with the other hand. The caveat is to slowly release the distal end of the stent first and then release the rest of the stent very carefully. After the stent was released at the exact desired location, the delivery device was retrieved with a recovery line being set about 50 cm from the upper-end edge of the stent. Once the stent was placed successfully, the traction line was taken out through the nasal cavity and suspended on the auricle to either facilitate the removal of the stent in case of any accidents or to timely adjust the stent to fulfill the treatment objective. Finally, the gastroscope was inserted again to visualize the expansion of the stent and a gastric tube or a nasojejunal tube retained if necessary. Figure 2 shows an example of the procedure.

### Postoperative management

After stent placement, a chest radiograph was taken on the same day to determine whether the stent had stayed open and that its position had remained unchanged. Feeding was started within 12 h after surgery, the children gradually progressed from liquids to semisolids (Dysphagia score III to II) while about 3 days later, most children could eat some solid food (Dysphagia score I)^13,15^. All the patients were instructed to chew slowly with small bites and to avoid rough, pungent or sticky food, and in particular were prohibited from eating cold food. A chest radiograph was taken at least once per month to visualize the position of the stent.

To determine whether an in-place stent should be removed or not, the following criteria were adopted: (1) the stent could be removed if the children couldn't tolerate chest pain or recurrent vomiting; (2) the stent must be removed if obvious tissue overgrowth or ulceration existed at the upper and/or lower edges of the stent during endoscopic surveillance; (3) the stent should be removed if the stent had been in position for ≥ 12 weeks; (4) the stent could be adjusted when it shifted from the original position, or the stent was removed when the stricture did not improve.

### Ethics approval and consent to participate

Our study was approved by the Ethics Committee of Shanghai Children's Hospital.

## Results

### Clinical features of pediatric patients with RBESs

**Figure 3:**
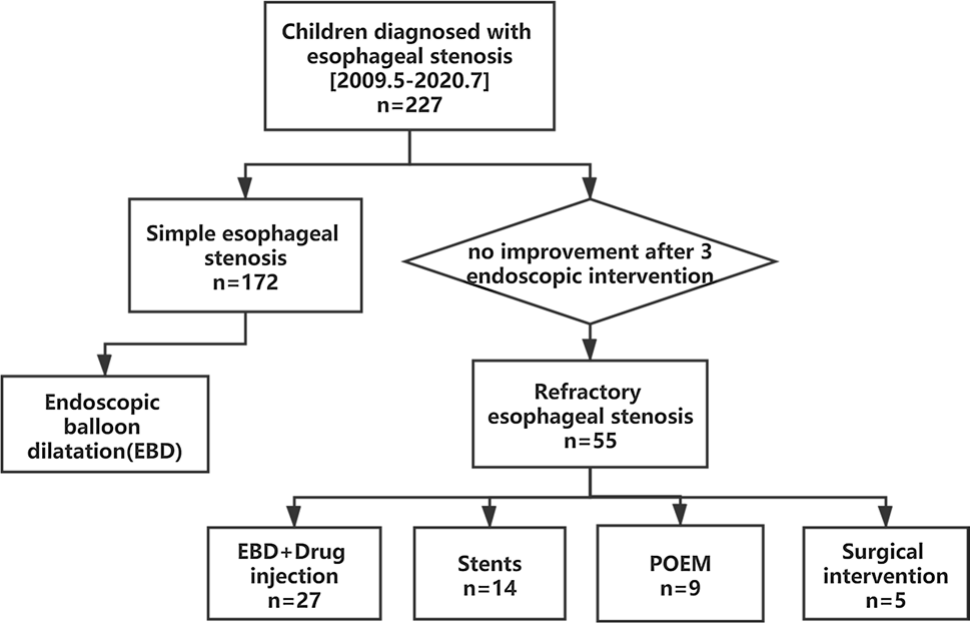
A flow diagram of children diagnosed with esophageal stenosis who required treatments.

From May 2009 to July 2020, a total of 14 children underwent FCSEMS placements, including 8 males and 6 females (age range 4 to 11 years) (Fig. 3). The children in this group had varying degrees of dysphagia, including 9 cases of grade III and 5 cases of grade IV of Ogilvie & Atkinson scores. The stenosis of the esophagus was mostly located in the middle and lower part of the esophagus with a length between 2 and 5 cm and a diameter between 2 and 5 mm (Table 1).

**Table 1:** Clinical characteristics of 14 pediatric patients with RBESs.

Case/gender/age (year)	Etiology of esophageal stricture	Stricture type	Prior therapy	Distance from incisors (cm)	Length of stricture (cm)	Diameter of stricture (mm)	Dysphagia grade
1/male/4	Caustic (acid)	Middle and lower esophagus, short segment	3 dilations	16	2.5	2	IV
2/female/6	Caustic (acid)	Middle and lower esophagus, short segment	4 dilations	17	3	3	III
3/male/5	Caustic (alkali)	Middle and lower esophagus, short segment	5 dilations	16	3	2	III
4/male/6	Caustic (alkali)	Lower esophagus, long segment	4 dilations	18	3.5	3	IV
5/male/11	Esophageal stricture after leukemia chemotherapy	Lower esophagus, long segment	5 dilations	20	4	3	IV
6/female/6	Caustic (alkali)	Middle and lower esophagus, short segment	4 dilations	15	3	3	III
7/female/8	Caustic (acid)	Lower esophagus, long segment	6 dilations	18	3.5	4	III
8/male/7	Caustic (acid)	Lower esophagus, short segment	4 dilations	20	2.5	3	III
9/female/8	Caustic (alkali)	Middle and lower esophagus, long segment	8 dilations	18	4	2	IV
10/male/5	Caustic (acid)	Lower esophagus, long segment	3 dilations	17	3	2	IV
11/male/6	Achalasia of cardia	Cardia, short segment	7 dilations	23	2	3	III
12/female/5	Achalasia of cardia	Cardia, short segment	4 dilations	21	2	3	III
13/female/7	Esophageal perforation after POEM	Lower esophagus, long segment	3 dilations	22	4	4	III
14/male/7	Esophageal perforation after endoscopic balloon dilation	Lower esophagus, long segment	3 dilations	19	5	5	III

RBESs, refractory benign esophageal strictures.

**Figure 4:**
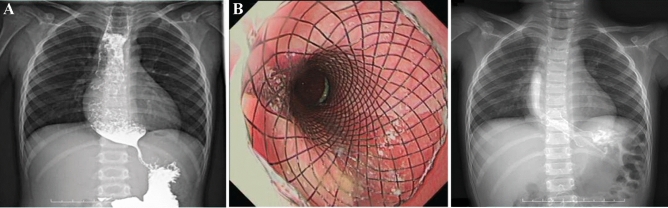
Stent placement in a patient with achalasia of cardia (case 12). (**A**) The upper gastrointestinal tract radiography showed a beak-like change, suggesting it was difficult for the barium to pass through before surgery; (**B**) A stent was placed and expanded as the patient body temperature rose; (**C**) Upper gastrointestinal tract radiography showed normal passing through of the contrast agent after stent placement.

**Figure 5:**
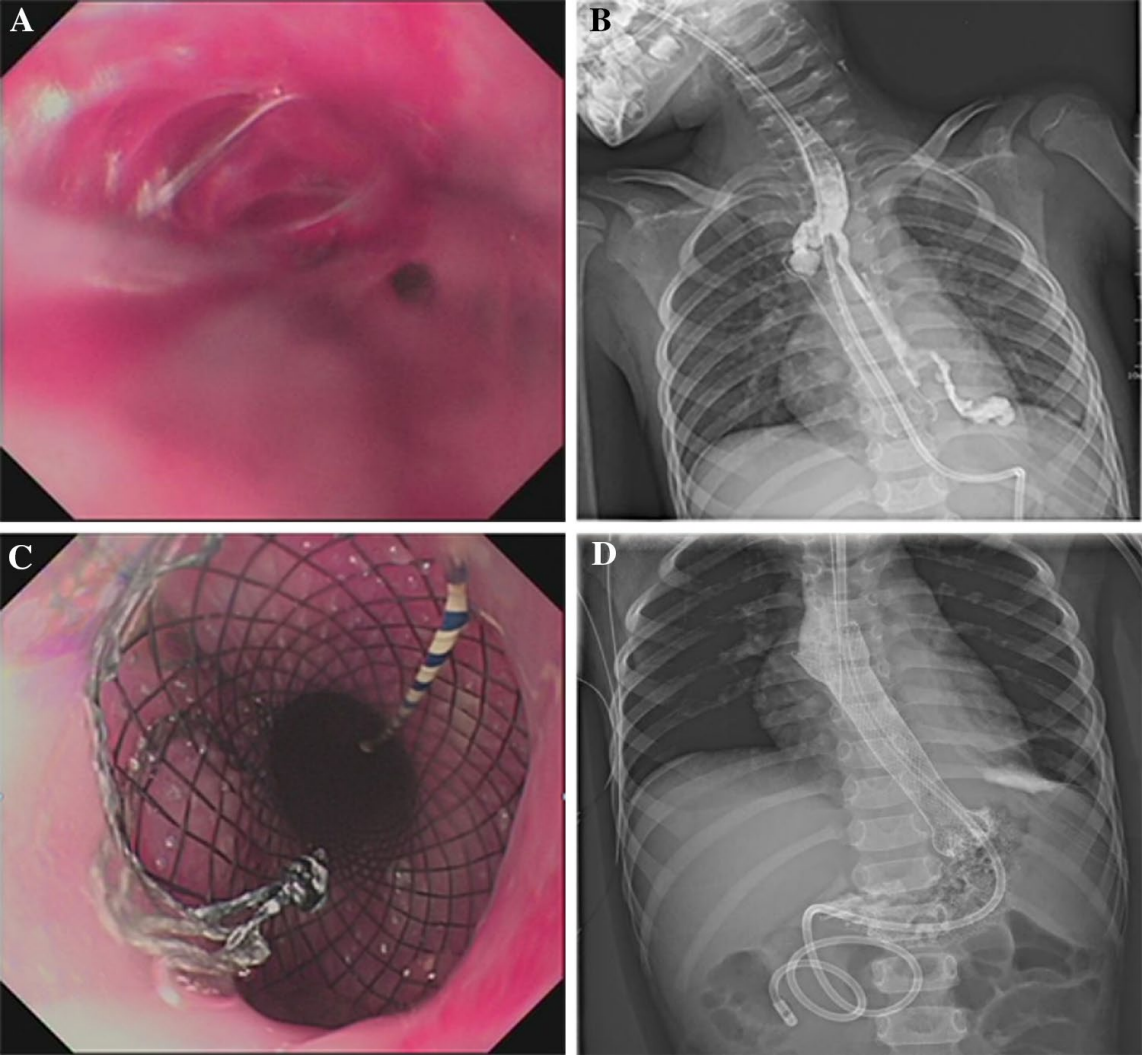
Stent placement in a patient with an EBD-induced esophageal perforation (case 14). (**A**) Esophageal perforation secondary to endoscopic balloon dilatation, the upper oral mucosa of the stricture was torn and bleeding; (**B**) Stent malapposition occurred 5 days after the first stent implantation; upper gastrointestinal tract radiography revealed that contrast medium infiltrated the mediastinum; (**C**) The original esophageal stent was replaced by a redesigned stent with larger upper bell mouth; (**D**) After the second esophageal stent was placed, upper gastrointestinal tract radiography revealed that the fistula was plugged, without contrast medium extravasating.

**Figure 6:**
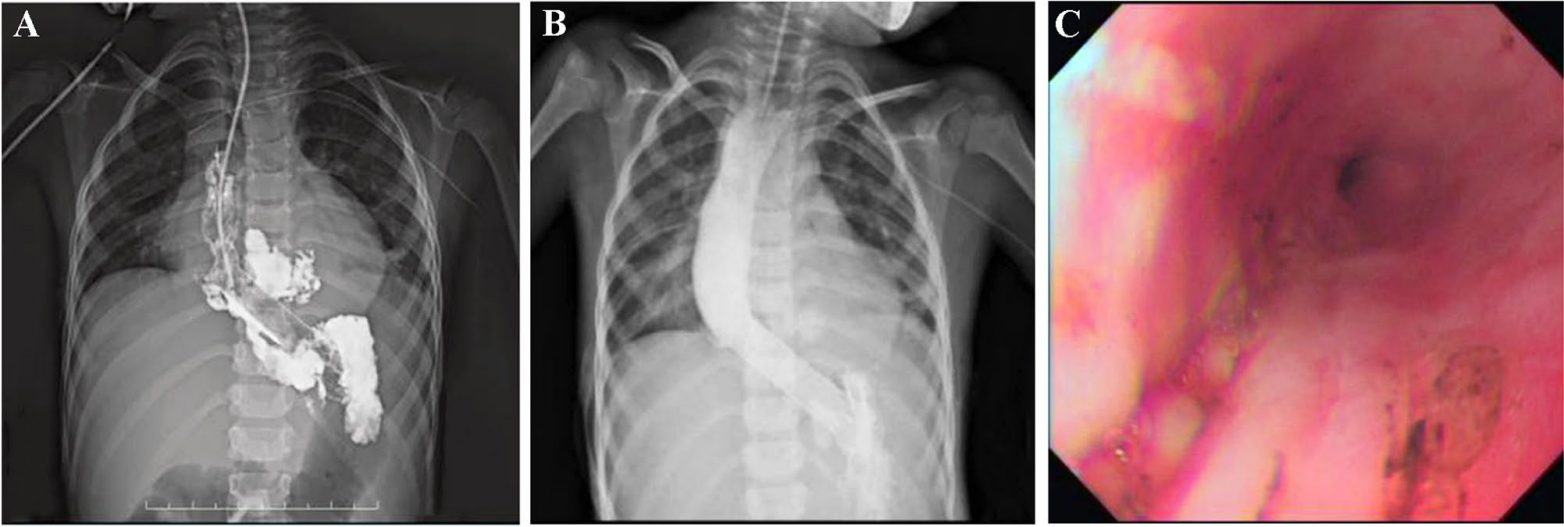
Stent placement in a patient with esophageal perforation after POEM (case 13). (**A**) A stent was placed after esophageal perforation was found during POEM, but stent migration occurred after 7 days; (**B**) After the stent position was adjusted under the endoscope, gastrointestinal tract radiography did not reveal extravasation of contrast medium; (**C**) The fistula was closed after the stent was removed 1.5 months later.

The esophagus of all 14 pediatric patients were imaged using contrast upper gastrointestinal tract radiography, of whom 10 with chemically corrosive esophageal stricture showed that the contrast medium passed through the esophagus slowly and unveiled one or more segments of stenosis along the course of the esophagus, with dilated portions at the upper part of the esophagus, 2 cases of achalasia of cardia featured the stenosis of the lower esophagus as unique beak-like changes with the difficulty in passing the contrast medium (Fig. 4A), 1 case of esophageal perforation caused by balloon dilatation of esophageal stricture (Fig. 5A) showed that the contrast medium had infiltrated the mediastinum (Fig. 5B), and 1 case had esophageal perforation after peroral endoscopic myotomy (POEM) (Fig. 6A).

### Treatment outcome and complications

Under gastroscopy, FCSEMSs were successfully implanted in 14 patients, among whom 9 patients were stented once, 4 twice, and 1 three times, thus a total of 20 stents were placed. During the follow-up period from 5 to 83 months, except a 4-year-old child whose stent was removed 36 h after the placement due to intolerance, dysphagia was significantly alleviated in the remaining 13 patients with the Ogilvie & Atkinson scores improved from grade III-IV to grade 0-I, and the diameters of the stenosis was expanded from 2–5 mm to 9–14 mm after the stents were placed (Table 2).

**Table 2:** Treatment outcomes and complications after stent placement.

Case/gender/age (year)	Number of stent placement	Stent model (mm × mm)	Time of stent retention (day)	Complications/recurrence	Treatment	Follow-up (month)	Diameter of stricture (mm)	Dysphagia grade
1/male/4	1	12 × 55	1.5	Obvious chest pain	Stent removal, balloon dilation * 5	16	5	II
2/female/6	1	14 × 60	52	Vomiting	Acid inhibition, etc	18	10	I
3/male/5	1	12 × 60	50	–	/	22	9	0
4/male/6	1	14 × 70	60	–	/	5	12	0
5/male/11	3	14 × 75 (1) 16 × 70 (2) 16 × 65 (3)	95/63/49	Recurrence 3 months later: inflammatory granulation tissue hyperplasia	Stent * 3 + balloon dilation * 2	83	12	I
6/female/6	1	14 × 60	55	–	/	26	11	0
7/female/8	2	14 × 70 (1) 16 × 70 (2)	45/52	–	/	35	11	I
8/male/7	1	14 × 60	57	–	/	42	11	I
9/female/8	2	12 × 75 (1) 14 × 70 (2)	88/62	Stent embedding caused by reactive tissue overgrowth, recurrence 3 months later	Stent * 2 + balloon dilation * 3	33	12	I
10/male/5	2	12 × 70 (1) 14 × 65 (2)	57/41	Recurrence 3 months later	Stent * 2 + balloon dilation * 1	41	10	I
11/male/6	1	14 × 60	45	Mild chest pain	/	23	12	0
12/female/5	1	12 × 55	50	–	/	18	10	I
13/female/7	1	16 × 65	46	Stent migration	Adjust the stent under endoscope	15	13	I
14/male/7	2	16 × 90 (1) 16 × 105 (2)	5/56	Stent malapposition	Reposition the newly designed stent	65	14	0

–, no complication and recurrence. /, no treatment.

**Figure 7:**
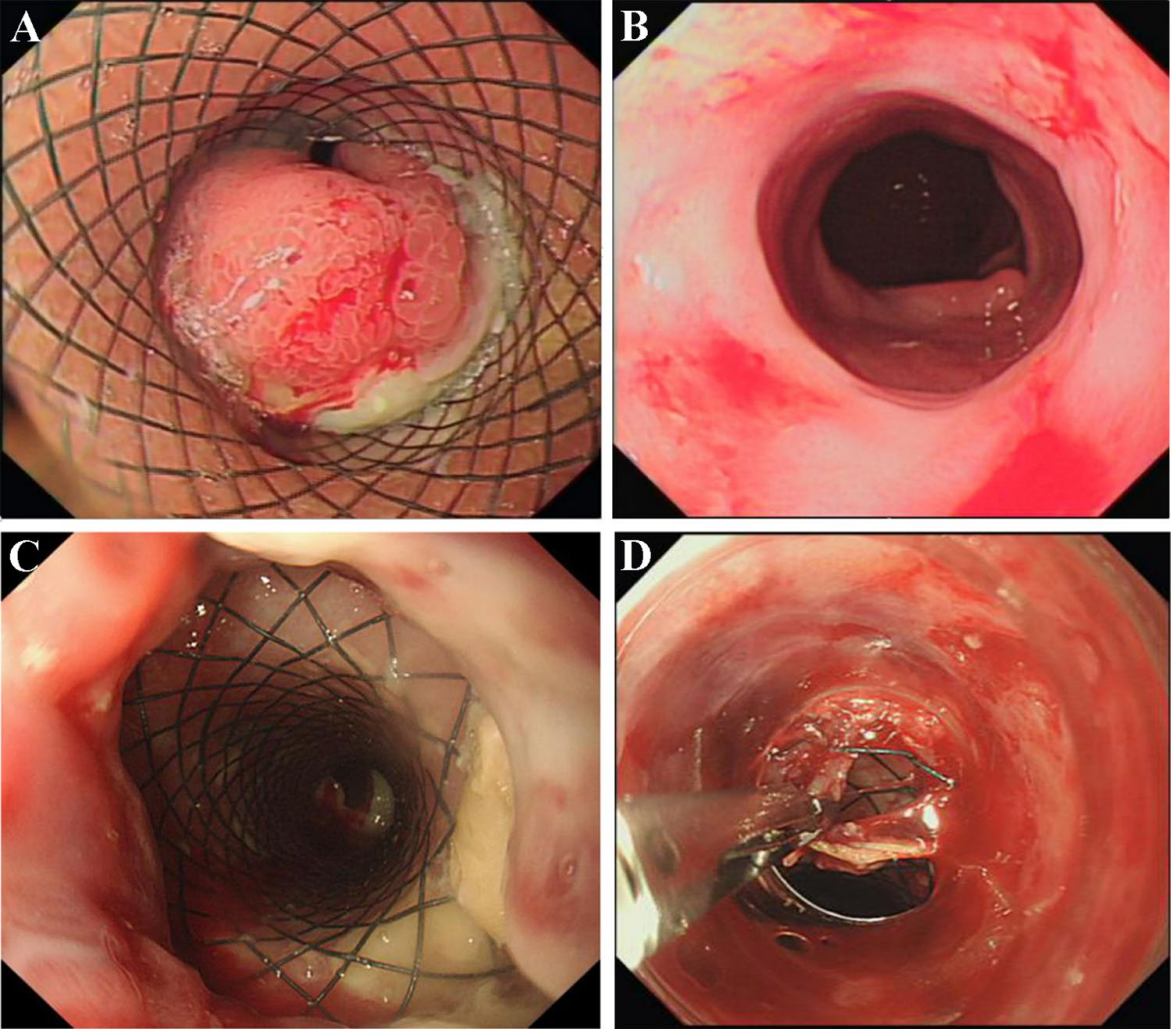
Management of adverse events related to long-term stent placement (cases 5 and 9). (In case 5) (**A**) Reactive inflammatory granulation tissue hyperplasia was seen at the bottom of the stent; (**B**) The esophageal lumen was changed following the removal of the third esophageal stent, the esophageal diameter was significantly enlarged without inflammatory granulation tissue hyperplasia noticed; (In case 9) (**C**) Stent embedding was caused by reactive tissue overgrowth; (**D**) The stent was not able to be removed on the first attempt but was successfully removed by biopsy forceps without perforation and active bleeding after part of the granulation tissue was excised.

Two patients with achalasia of cardia (cases 11 and 12) showed remarkable improvement in the symptoms of vomiting (Fig. 4B,C) without the need for further stent placement. Two patients (cases 2 and 11) presented symptoms of vomiting and chest pain 2–3 days after stenting, which were relieved by conservative management without stent removal. Three patients (cases 5, 9 and 10) developed restenosis 3 months after stent removal, but the degree of stenosis had been significantly mitigated compared to before stenting and the lumens at the narrowing were significantly enlarged, with improvement in swallowing difficulty after the new stents were placed (Fig. 7B).

More specifically, 2 patients (cases 5 and 9) failed to meet the follow-up schedules due to parental reasons, but were subsequently readmitted for chest pain and discomfort symptoms that occurred again about 3 months after stent placement. To treat these patients, their stents were removed after the finding of inflammatory granulation tissue hyperplasia at the lower end of the stent in case 5 (Fig. 7A) and stent embedding in case 9 (Fig. 7C). Fortunately, the embedded stent was removed successfully with the help of biopsy forceps (Fig. 7D), but both cases still needed additional stent and balloon dilatation treatment before their symptoms were completely relieved.

The child with esophageal perforation after POEM (case 13) was given an esophageal stent (Fig. 6A), but 7 days after its placement, the patient developed stent migration, which was dealt with by an adjustment of the stent position under endoscopy, consequently leading to the closure of the fistula one and half months later (Fig. 6B,C). The child with esophageal perforation (case 14) underwent closed thoracic drainage for esophageal mediastinal fistula and then received empyema exploration, with esophageal stent placement. However, the patient was found to have stent malapposition 5 days after the operation (Fig. 5B), which required the removal of the original stent and its replacement with an esophageal stent with a larger upper bell mouth (Fig. 5C,D). Two months later, the stent was removed, the fistula healed and the stenosis disappeared.

## Discussion

Currently, FCSEMS has not been designed for pediatric use, thus needs to be further customized stent development for pediatric use according to the uniqueness of the esophageal stenosis or stricture in children. The present study showed that the success of stent placement was closely associated with the radial support performance of the stent, which mainly depends on the number of heads, wire diameter and lead distance of the stent^14^. Through preoperative imaging, the stent customization company deduced suitable parameters for FCSEMS for use in the pediatric esophagus as follows: the number of the head was 22 or 24, wire diameter between 0.18 and 0.22 mm and lead distance ranging from 60 to 70 mm. In our study, all the 14 patients had 22 for the head number of stents, 0.18 to 0.20 mm for the diameter of steel wire and 63.0 ± 2.5 mm for the lead length. Additionally, the length of the stent was advisably designed to be 10–20 mm longer than the narrowing at both the upper and lower ends. Taking the length of the stenosis segment into consideration, the stent lengths in our 14 patients ranged from 55 to 105 mm.

Moreover, the stent diameter was designed in accordance with the size of the inner diameter of the stenosis segment. For pediatric patients with aged > 5 years, the stent diameter ranges from 12 to 16 mm, but if children presented with esophageal perforation, the stent diameter was made slightly larger. The stent diameter was chosen in this study to be 16 mm for cases 5, 7, 13 and 14. Our knowledge here is that it is necessary to adjust timely the length and diameter of the stenosis according to findings shown in the preoperative upper gastrointestinal tract radiography. The diameter of the stent may be selected from small to large to avoid the extrusion of the esophageal wall and its adjacent organs.

Nitinol memory alloy was chosen as the stent wire coating material as it shrinks when exposed to cold and expands when exposed to heat. After placement, the stent can gradually and continuously expand as the patient's body temperature rises, thus avoiding direct mechanical expansion and significant irritation to the esophageal wall. The outer stent was covered with a full silica gel film, which may account for the functions of sealing the fistula and compressing hemostasis. The two ends of the stent were attached with 2–5 mm long silicone membrane soft skirts, which further reduced the pressure on the esophageal wall at both ends of the stent. For gastroesophageal reflux that may occur after stent placement in patients with achalasia of the cardia, a reflux prevention valve should be designed on the lower edge of the esophageal stent. The upper end of the stent is provided with a recovery line to facilitate its removal or to make timely adjustments to its position.

To prevent effectively the displacement of the stent, the upper and lower ends of the stent were designed with a bell mouth. Generally, the diameter of the bell mouth is larger than that of the stent (5.5 ± 0.5 mm), but for the stenosis of the middle and lower segment, especially the stenosis near the second esophageal narrowing, the design of the upper bell mouth diameter should not be large enough to cause a risk of cardiovascular accidents. Additionally, the length and diameter of stents for multiple treatments in the same child are usually different, and should be adjusted according to the changes of the stenosis length and diameter. In our experience, it is better to place stents multiple times rather than to pursue one action for the prospective clinical outcome.

The present study showed that 13 of 14 pediatric patients with RBESs (92.8%), who either had failed to obtain sustained symptomatic relief after more than 3 sessions of dilations or developed balloon dilation related complications, successfully had FCSEMS placed with long-term symptomatic improvement over a follow-up period of 5 to 83 months. Three mechanical features of FCSEMS contributed to this satisfactory outcome: First, the tailored stent follows the physiological structure of the esophagus and the special pathological manifestations of benign stenosis; Second, slow expansion of the metal stent with the uniform transmission of radiation force to the esophageal wall causes slow tear and slow scar healing in the regular muscle layer, subsequently making restenosis less likely to occur; and third, the FCSEMS placement prevents scar contraction and tissue adhesion in the process of healing, which stimulates less edema formation than balloon dilatation^3^.

Currently, the indication for using FCSEMS for benign stenosis of pediatric patients still remains undefined. However, esophageal stent placement should not be offered to pediatric RBES patients with the disease conditions of high anastomotic stenosis, stent migration and abscission, retrosternal pain, and other factors that result from the relatively fast peristalsis of the esophagus. In addition, the placement of an esophageal stent over anastomotic stenosis that passes behind the aortic arch may produce devastating complications and so is a high-risk procedure, leading to unsuitability for stent placement^16^. Moreover, stent placement should not be offered to patients with either esophageal anastomotic or corrosive stenosis of a length < 3 cm, which could likely be successfully dilated. Taken together, stent placement should mainly be applied to children with congenital, corrosive and postoperative anastomotic stenosis due to repeated dilatation failure^17^. Our current retrospective analysis posted several main indications for esophageal stenting on our patients thus: (1) Short-to-medium stenosis located in the middle and lower esophagus with repeated stenosis over a short period after more than three balloon dilatations; (2) If the esophageal stenosis had a balloon dilatation treatment in the early stage, the post-dilation diameter of the stenosis should be > 5 mm; (3) For children with achalasia of the cardia, the lower end of the stent needs to be designed with an anti-reflux valve to reduce gastroesophageal reflux; (4) Children who had esophageal perforation or esophageal fistula.

Since a permanent stent is not an appropriate choice for a pediatric esophagus, the FCSEMS should be retrieved after being placed for a certain period of time. However, until now, no conclusion has been made on the optimal time for a stent to be placed in the esophagus of children. Best et al. suggested at least two weeks^2^, while other studies reported various durations of esophageal stent implantation from 40 days to 8 months for good clinical effectiveness^18,19^. If a stent has only been placed in the esophagus for a short period, the scar tissue in the stenotic segment may not be stiff enough fully to expand the stenosis, while if too long, both ends of the stent will induce inflammatory polyps, which may increase the chance of re-stenosis^20^. Our experience of 14 children with RBES taught us that the esophageal lodging time of a stent should optionally be between 6 and 8 weeks, with a maximum of 12 weeks and its position adjusted according to the follow-up assessment of its position.

One of the most common complications after stent placement is chest pain, which often subsides within one week without the need for administration of analgesics. However, this post-stenting condition red-flags the possibility of other complications such as bleeding, pneumothorax and perforation, so should be vigorously investigated to prevent any devastating event^21^. In our study, intolerable post-stenting chest pain in a 4-year-old child, who was the youngest one among all the patients in the study, was relieved only after the stent was removed 36 h after its placement, thereby suggesting that the age of stent placement should not be in very young patients. For our practice, we set 5-years old as the minimum age requirement for the use of FCSEMS to avoid poor compliance and difficulty in postoperative care.

After stenting, the stent position can adjust under endoscopic control. Studies in the literature have reported that the incidence of adult esophageal stent displacement was 37–63%^22,23^, and suggested the smaller the inner diameter or the closer the stent was placed to the upper or lower end of the esophagus, the more likely it was to shift position^24^. In our study, only 1 child (case 13) had stent migration 7 days after stent placement (1/14 7.1%), and we believe that the accurate evaluation of stenosis and the reasonable and scientific design of the stent is important, while special attention should be paid to the size of the upper bell mouth. Moreover, as self-expandable stents, accurate stent placement, rigorous diet management and postoperative care of the children play important roles in keeping the stent in position.

Another issue that may affect the long-term outcome after FCSEMS placement is whether the stent perfectly fits the esophageal wall. In case 14 of this study, the stent was designed for the first time for children with EBD-induced esophageal perforation, which should be treated at an early stage to minimize contamination outside the esophageal cavity and restore the integrity of the lumen as soon as possible^25^; however, it failed fully to fit and engage with the esophageal wall. After enlarging the upper bell mouth and skirt, the stent effectively closed the fistula, and 2 months later, the reexamination after the stent was taken out showed that the fistula was healed and the original esophageal stricture was relieved.

The stent should be removed as soon as therapeutic effect is achieved, otherwise, it may increase the risk of inflammatory polyp hyperplasia and stent embedding, which can result in complications and difficulties in stent removal and even result in perforation of the esophagus. In case 5, the stent was in place for 95 days, causing hyperplasia of inflammatory granulation tissue at the bottom of the stent, while in case 9, stent embedding caused reactive tissue overgrowth after 88 days of stent implantation. It has been reported that children with stent placement are more likely to have inflammatory polyp hyperplasia^26^ for the following reasons: first, inflammatory polyps might be caused by the stent as a pro-inflammatory stimulus to the esophageal wall mucosa; second, because of esophageal peristalsis, the stent will repeatedly rub the mucosa of the esophageal wall, and especially the expansion of the bell mouth at both ends could produce a transverse cutting force with the flexible esophagus, resulting in mechanical injury of the esophagus; third, if the stent implantation is too long, gastric acid reflux may corrode the esophageal mucosa, leading to granulation tissue hyperplasia^27^. The enlarged inflammatory polyps usually do not need special treatment and tend to disappear after the pro-inflammatory stimulus is removed.

Restenosis after stent removal is a long-term complication for stent placement. One study reported that the restenosis rate of a "Z" stent after esophageal stent placement was 27% in adult patients^28^, while another study on the application of Z-stents to treat 15 children with corrosive esophageal stenosis found 8 patients (53.3%) had restenosis during a 3-month follow-up^19^. In contrast, the present study revealed that 3 of 14 children had restenosis after stent removal (21.4%), which was similar to the reported rate of 20.8%^26^, but better than that in the two studies documented above. However, stenosis after stent placement was significantly reduced compared with before stenting. Stents were placed again based on the clinical symptoms and imaging findings, and the postoperative follow-up showed satisfactory results.

Limitations of the current study include its retrospective study design and the small patient number. Although our endoscopic center treats many children with esophagus disorders every year, the number of children diagnosed with refractory esophageal stenosis is still not very large. Further prospective studies with larger sample sizes are needed to validate the present findings.

## Conclusions

In summary, the use of FCSEMS for RBES is safe and feasible in pediatric patients. The age, stenosis characteristics and esophageal anatomical structure should be fully considered before implementing the stenting procedure. The FCSEMSs should be customized based on upper gastrointestinal tract radiography or chest CT scans of the pediatric patients. Timely management of complications after stent placement may safeguard its long-term effectiveness.

## Data Availability

The datasets generated during and/or analysed during the current study are available from the corresponding author on reasonable request.
